# 

**Published:** 1899-11-04

**Authors:** 


### NOTICE TO CORRESPONDENTS.

All MS., Letters, Books for Review, and other matters intended for the Editor should be addressed THE EDITOR, "THE HOSPITAL," THE HOSPITAL BUILDING, 28 & 29, SOUTHAMPTON STREET, STRAND, W.C.
The Editor cannot undertake to return rejected MS., even when accompanied by stamped directed envelope.

**Notice to Advertisers and Subscribers.**

All Advertisements, Orders for Copies of THE HOSPITAL, and Business Communications should be addressed to **THE MANAGER (Not to the Editor),** THE HOSPITAL, The Hospital Building, 28 & 29, Southampton Street, Strand, London, W.C.
The Cost of Subscription, post free, is as follows:—Per week, 2½d.; per quarter, 2s. 8d.; per half-year, 5s. 3d.; per annum, 10s. 6d. Foreign:—Per annum, 15s. 2d., payable, in all cases, in advance.

**NOTICE.—Vol. XXVI. of "The Hospital" (April, 1899, to September, 1899), in handsome cloth binding, will shortly be on sale at the Office of this Journal, price 7s. 6d. Cases for binding the Numbers contained in this Volume 1s. 6d. Index to Vol. XXVI., 2½d., post free.**
**The Monthly Part for November is now ready. Price 6d., by post 8d.**

### BOOKS RECEIVED.

P. S. King and Son.

"How the English Workman Lives." By a German Coal Miner (Ernst Dückershoff).

J. and A. Churchill.

"The Schott Methods of Treatment of Chronic Diseases of the Heart." By W. Bezly Thorne, M.D., M.R.C.P.

"Selected Papers on Stone, Prostate, and other Urinary Disorders." By Reginald Harrison, F.R.C.S.

L. Bruck.

"On the Uses and Abuses of Public Hospitals." By L. Bruck.

University Corr. Coll. Press.

"London University Guide for 1899-1900."

### Scraps and Gleanings.

THE question of the proposed Dollar Infectious Hospital is now in abeyance till next month's municipal election, when a decision on this point will be come to.

THE number of patients admitted into the Herts Convalescent Home during the past year was 741, making a total of 10,961 since the charity was established 23 years ago.

THE Aberdeen Hospital Saturday Fund has this year amounted to £1,300; this is the first year that this fund has had a house-to-house collection, which realised £551 odd; the sports realised £345 odd.

A COMMITTEE has been appointed to present an appeal to the public on behalf of the Stockport Infirmary Building Fund, which is in the position of having only £107 odd on hand to meet possible requirements of £4,000.

SINCE 1870 the collections in aid of the Newcastle medical charities by the Hospital Sunday Fund amount to a sum of over £107,000. In 1870, the first year of its establishment, the amount collected was £1,992, while in 1898 it was £4,472.

IT is stated that the Stroud Joint Hospital Board have decided upon a site in the populous district known as Horns Road for the erection of an infectious diseases hospital. This proposal has already met with some opposition on the part of ratepayers.

IT is proposed, in order to celebrate the centenary of the Plymouth Public Dispensary, that an appeal be made to the public of Plymouth to wipe off the existing debt of £219, and so enable this institution to start fresh after 100 years' work with a clean slate.

DURING the past year 3,670 patients were treated at Plymouth Public Dispensary, as against 3,345 last year. At a cost of £515 the buildings are to be enlarged and the plans have already been approved. The money for this extension has been provided by selling out investments.

THE revenue for the last half-year of the North Riding Infirmary was £1,718 odd, as against £1,703 odd for the corresponding half of the previous year. The expenditure had also increased, owing, it was stated at the last half-yearly meeting, to the extended accommodation by means of which it was possible to provide extra service.

### The Student of Medicine.

**APPOINTMENTS.**

J. A. BALCK, M.B., Ch.B.Edin., appointed House Surgeon to Royal Hants County Hospital, Winchester. L. J. Godson, M.R.C.S.Eng., L.R.C.P.Lond., appointed Surgeon to the Salop Infirmary. W. J. Harris, M.D.Cantab., M.R.C.P., appointed Assistant Physician to the City of London Hospital for Diseases of the Chest, Victoria Park, E. E. W. Martin, M.B., Ch.B., appointed Junior House Physician to the City of London Hospital for Diseases of the Chest, Victoria Park, E. E. J. F. Moore, M.R.C.S.Eng., L.R.C.P.Lond., appointed Medical Officer to the Workhouse of the Bethnal Green Union. J. P. Parkinson, M.D., M.R.C.P.Lond., F.R.C.S.Eng., appointed Visiting Physician to the London Temperance Hospital. W. J. Potts, M.D. Lond., M.B., appointed Medical Superintendent of the new Infirmary, Bethnal Green. F. W. Price, M.B., C.M.Edin., appointed Assistant Resident Medical Officer to the Brompton Hospital for Consumption. G. A. Rorie, M.B.Edin., appointed Junior Assistant Medical Officer in the Cumberland and Westmoreland Asylum, Carlisle. J. Stewart, J.P., L.R.C.P., L.R.C.S.Edin., appointed Certifying Factory Surgeon for the Burgh of Inverary, and the Civil Parish of Inverary, in the Mid-Argyll District of Argyllshire. M. Thorne, L.S.A.Lond., M.D.Brux., appointed Teacher on the Theory and Practice of Vaccination by the Local Government Board. O. K. Williamson, M.A., M.B., B.C.Cantab., M.R.C.S.Eng., L.R.C.P.Lond., appointed Pathologist to the City of London Hospital for Diseases of the Chest, Victoria Park, E.

Accouchement Requisites. Ambulance Cases.

**NOW READY,**

A REPRINT OF

### A NURSING HANDBOOK

TOGETHER WITH S. MAW, SON, AND THOMPSON'S

### COMPLETE CATALOGUE OF NURSING REQUISITES.

The above work is now republished at a cost of 1s. per copy. Messrs. S. M., S., and T. have been compelled to make this charge on account of the extreme popularity of the work and the great expense incurred in its production.

***Post Free on receipt of 1s.***

### S. MAW, SON, and THOMPSON,

7 to 12, ALDERSGATE STREET, LONDON, E.C.

